# Update on Edoxaban for the Prevention and Treatment of Thromboembolism: Clinical Applications Based on Current Evidence

**DOI:** 10.1155/2015/920361

**Published:** 2015-08-16

**Authors:** Ali Zalpour, Thein Hlaing Oo

**Affiliations:** ^1^University of Texas MD Anderson Cancer Center, 1400 Pressler Avenue, Unit 1465, FCT 13.5021, Houston, TX 77030, USA; ^2^Section of Thrombosis & Benign Hematology, The University of Texas MD Anderson Cancer Center, Houston, TX, USA

## Abstract

Vitamin K antagonists (VKA) and heparins have been utilized for the prevention and treatment of thromboembolism (arterial and venous) for decades. Targeting and inhibiting specific coagulation factors have led to new discoveries in the pharmacotherapy of thromboembolism management. These targeted anticoagulants are known as direct oral anticoagulants (DOACs). Two pharmacologically distinct classes of targeted agents are dabigatran etexilate (Direct Thrombin Inhibitor (DTI)) and rivaroxaban, apixaban, and edoxaban (direct oral factor Xa inhibitors (OFXaIs)). Emerging evidence from the clinical trials has shown that DOACs are noninferior to VKA or low-molecular-weight heparins in the prevention and treatment of thromboembolism. This review examines the role of edoxaban, a recently approved OFXaI, in the prevention and treatment of thromboembolism based on the available published literature. The management of edoxaban in the perioperative setting, reversibility in bleeding cases, its role in cancer patients, the relevance of drug-drug interactions, patient satisfaction, financial impacts, and patient education will be discussed.

## 1. Introduction

Unfractionated heparin (UFH), a highly sulfated naturally occurring glycosaminoglycan, was discovered in Howell's laboratories in the early 1920s. Sweet clover disease or hemorrhagic disease of the cattle in Wisconsin led to the discovery of coumarin. Since then, warfarin has become one of the mostly used antithrombotic agents [1]. Low-molecular-weight heparin (LMWH) was also discovered in the late 1970s and early 1980s as clinicians sought longer acting heparins with a more predicable pharmacokinetic profile. UFH requires frequent monitoring and administration in a hospital setting and carries a risk of heparin-induced thrombocytopenia (HIT). Warfarin demonstrates unpredictable pharmacodynamic (PD) and pharmacokinetic (PK) properties and numerous drug-drug and drug-food interactions and requires frequent international normalized ratio (INR) monitoring. In the past decade, an injectable factor Xa inhibitor, fondaparinux, was introduced. LMWH and fondaparinux exhibit a more predictable PK and PD profile, but patients are subjected to injections that can be burdensome [2].

Advances in pharmacology and drug design therapy have led to the development and introduction of DOACs such as dabigatran, rivaroxaban, apixaban, and edoxaban [3–5]. DOACs have been approved for the prevention of stroke in nonalular atrial fibrillation (NVAF) and the prevention and treatment of venous thromboembolism (VTE). Numerous trials have shown noninferiority of DOACs compared to standard-of-care (SOC) anticoagulants. DOACs have eased the burden of frequent monitoring and painful injections, curtailed food and drug interactions, reduced cost, and achieved higher degree of patient satisfaction [6, 7].

## 2. Physiology of Hemostasis and Pharmacology of Edoxaban

**Figure 1:**
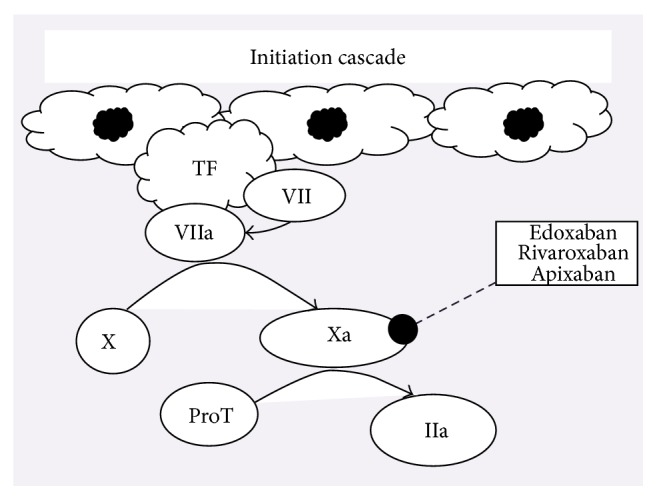
Adapted with permission: Zalpour and Oo [8]. Abbreviations: TF, tissue factor; VII, factor VII; VIIa, activated factor VII; X, factor X; Xa, activated factor X; ProT, prothrombin; IIa, thrombin; IX, factor IX; IXa, activated factor IX; Xa, activated factor X; Va, activated factor V; VIIIa, activated factor VIII; vWF, Von Willebrand factor.

**Figure 2:**
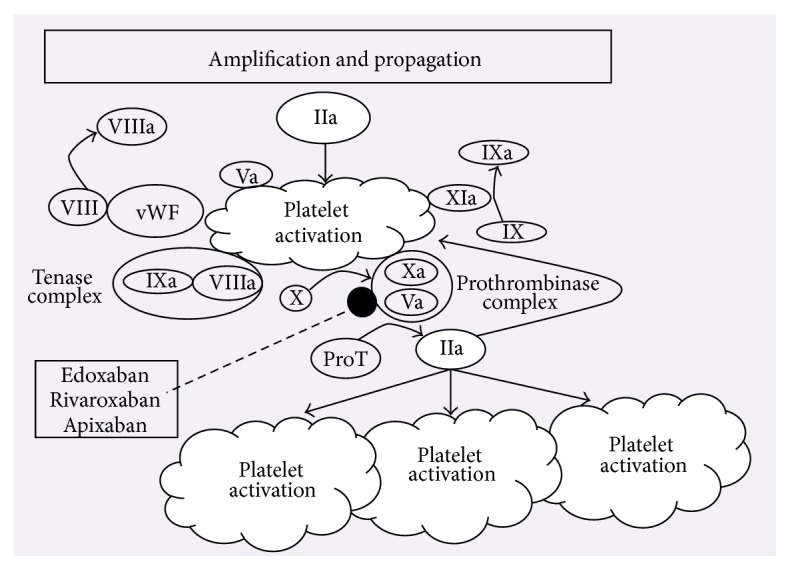
Adapted with permission: Zalpour and Oo [8]. Abbreviations: TF, tissue factor; VII, factor VII; VIIa, activated factor VII; X, factor X; Xa, activated factor X; ProT, prothrombin; IIa, thrombin; IX, factor IX; IXa, activated factor IX; Xa activated factor X; Va, activated factor V; VIIIa, activated factor VIII; vWF, Von Willebrand factor.

Coagulation cascade is a multistep interaction characterized by the sequential activation of coagulation factor proteins and their interactions with platelets [9]. Preserving hemostasis is an intricate process following the activation of intrinsic (contact activation) or extrinsic (tissue factor) pathways [10, 11]. The initiation phase of the coagulation involves the generation of tissue factor (TF) which subsequently leads to the activation of factors FVIIa and FXa and the generation of FIIa (thrombin). In the amplification and propagation phases, thrombin activates platelets and, in sequence, factors VIIIa and IXa. Platelet activation induces a surge in thrombin generation leading to the clot formation within the vasculature [12]. The vitamin K antagonist inhibits factors II, VII, XI, and X and proteins C, S, and Z [13]. Heparins inactivate FIIa and FXa via binding their saccharide chain to antithrombin (AT) [14]. FXa is considered a great target for inhibition, as one molecule of FXa can generate approximately 1,000 molecules of thrombin [15]. Edoxaban inhibits thrombin generation by actively inhibiting free and bound FXa in the prothrombinase complex. This inhibition leads to halting of positive feedback loop existing between FXa and FIIa (Figures 1 and 2). The capability of edoxaban to penetrate into the thrombus and rendering free and bound FXa inactive is proven to be beneficial, for the need for AT-drug complex is diminished [12].

## 3. Pharmacodynamics and Pharmacokinetics of Edoxaban

Edoxaban (molecular weight 838.274 gram/mol) exhibits a high affinity (>10,000-fold) to inhibit FXa without the need of binding to antithrombin and has a low affinity for FIIa [16]. Edoxaban is 55% protein bound and not completely removed by dialysis. The absorption of edoxaban from the gastrointestinal tract is about 60% and food has minimal effect on systemic exposure of area under the curve (AUC) [17]. Edoxaban reaches a maximum concentration in 1-2 hours and has a low volume of distribution of ~19.9 liters. In a phase 1 PK study of edoxaban, the *T*_1/2_ was 5.71 to 10.7 hours after a single dose administration ranging from 10 mg to 150 mg and 8.75 to 9.75 hours after multiple doses of edoxaban ranging from 90 mg to 120 mg daily [18]. The mean elimination half-life of edoxaban is estimated in the range of 10–14 hours and reaches a steady state concentration in 72 hours. Edoxaban is metabolized via hydrolysis with minimal enzymatic pathways of liver (CYP metabolism is less than 4%). Human carboxylesterase 1 (hCE-1) forms M4, a major metabolite of edoxaban, which is pharmacologically active. M4 reaches less than 10% of the exposure of the parent compound in healthy subjects. Exposure to the other metabolites of edoxaban is less than 5%. Edoxaban elimination is 50% via renal route and 50% via biliary and intestinal route [19]. Age has no direct effect on the PK of edoxaban; however, in patients with body weight less than 60 kilograms, there is an increase in exposure [20]. In a PK simulation study of 278 patients from phase 1 trials, the bioavailability (*F*) was estimated to be 67.2% in a dose ranging from 10 to 30 mg. Female patients exhibited 13.1% lower clearance (CL) than males. AUC_∞_ in females was higher than that in males as the dose of edoxaban increased from 10 mg to 180 mg; for example, in the edoxaban 90 mg, AUC_∞_ is 3,385 (ng*∗*h/mL) in male patients and 3,893 (ng*∗*h/mL) in female patients, respectively. The clinical significance of these findings has not been validated in clinical trials (Table 1) [21].

### 3.1. Pharmacodynamics and Pharmacokinetics of Edoxaban in Patients with Liver Dysfunction

The presence of encephalopathy or ascites (clinical parameters) along with serum albumin, serum bilirubin, and prothrombin time (laboratory parameters) collectively classifies patients into three distinct groups of liver diseases: Child-Pugh A (mild), Child-Pugh B (moderate), and Child-Pugh C (severe). Patients with Child-Pugh C class are typically excluded from the clinical trials involving anticoagulation due to excess bleeding risk [39]. In a hepatic impairment study (Child-Pugh A, *n* = 8; Child-Pugh B, *n* = 8) matched for healthy patients (*n* = 16), after the administration of single oral dose of edoxaban 15 mg, compared to healthy subjects, the AUC_∞_ decreased by 4.2% in subjects with Child-Pugh A and 4.8% in subjects with Child-Pugh B. The peak serum concentration (*C*_max_) decreased by 10% and 32% in patients with Child-Pugh A and Child-Pugh B, respectively [40]. Currently, no PK data exists for edoxaban use in patients with Child-Pugh C and the use of edoxaban in patients with moderate or severe hepatic impairment (Child-Pugh B and Child-Pugh C) is not recommended as these patients may have intrinsic coagulation abnormalities [17].

### 3.2. Pharmacodynamics and Pharmacokinetics of Edoxaban in Patients with Renal Dysfunction

Patients with end stage renal disease (ESRD) are typically excluded from the edoxaban clinical trials, because of increased bleeding risk. For patients with ESRD who may require anticoagulation, VKA and UFH are the anticoagulants of choice [41]. All OFXaIs have some degree of renal elimination: apixaban 27%, rivaroxaban 33%, and edoxaban 50%. OFXaIs are excluded in clinical trials enrolling patients with CrCl < 25–30 mL/min [42]. The safety and PK of edoxaban (15 mg, 30 mg, and 60 mg) daily for 12 weeks in patients with normal or mild renal insufficiency (CrCl ≥ 50 mL/min) and severe renal insufficiency (CrCl ≥ 15 to < 30 mL/min) in 92 patients have been evaluated. Patients on hemodialysis or at high risk for bleeding were excluded. The dose of edoxaban was decreased by 50% in patients with normal or mild renal insufficiency who weigh less than 60 kg or who are on verapamil or quinidine. The bleeding complication rates in patients with severe renal impairment (edoxaban 15 mg group) and normal or mild renal insufficiency (edoxaban 30 mg or edoxaban 60 mg group) were 20%, 23.8%, and 22.7%, respectively. This study showed that edoxaban 15 mg might be appropriate for patients with severe renal insufficiency [43].

In a PK study of 10 patients with ESRD on dialysis, patients received a single dose of edoxaban 15 mg in 2 different schemes: on-dialysis days, 2 hours before, and off-dialysis days between hemodialysis sessions. Patients with history of bleeding, major trauma, or major surgical procedure, peptic ulcer, or gastrointestinal bleeding within the past 6 months or those who use any drugs that are strong inhibitors or inducers of CYP3A4 or P-gp within the past 4 weeks were excluded. *T*_1/2_ was 10.6 ± 3.13 hours in on-dialysis group and 10.4 ± 2.72 in off-dialysis group. *T*_max_ on-dialysis and off-dialysis was 2.3 and 2.0 hours accordingly. The *C*_max_ was 53.3 ± 15.14 nanograms/milliliter (ng/mL) in on-dialysis group and 56.3 ± 23.25 ng/mL in off-dialysis group. The AUC_∞_ (ng*∗*L/hr) was 676.2 ± 220.86 in on-dialysis group versus 691.7 ± 149.84 in off-dialysis group. Clearance measured in (CL/h) was 24.7 ± 7.07 and 22.5 ± 4.50 in on-dialysis and in off-dialysis groups, respectively. These results showed that hemodialysis did not alter the PK of edoxaban. Mean percent of edoxaban protein binding remained ~60% in on- and off-dialysis groups. The effect of dialysis on M-4 (active metabolite of edoxaban) on *C*_max_ in on-dialysis and off-dialysis was 9.8 ± 7.05 ng/mL and 10.2 ± 4.98 ng/mL, respectively. The *T*_max_ was prolonged in off-dialysis group to 4 hours versus 2.1 hours in on-dialysis group and the AUC_*∞*_ was lower in off-dialysis group versus on-dialysis group, 151.9 ± 101.843 (ng*∗*L/hr) versus 193.3 ± 255.48 (ng*∗*L/hr). The *T*_1/2_ was prolonged at 8.05 to 17.95 hours in on-dialysis group versus 7.52 to 15.08 hours in off-dialysis group. There was no bleeding, death, or any serious adverse reactions. This study showed that dialysis has no effect on the removal of edoxaban [44]. Currently, there are no clinical trials comparing different does of edoxaban in patients with renal insufficiency; however, a recent metaregression analysis was undertaken to examine the safety and efficacy of DOACs versus warfarin in patients with various degrees of renal function. The hazard ratio (HR) of bleeding in patients with moderate renal impairment (CrCl 25–49 mL/min), mild renal impairment (CrCl 50–79 mL/min), and nonrenal impairment (CrCl > 80 mL/min) has been estimated for edoxaban 30 mg versus warfarin as HR: 0.31 (95% Confidence Interval (CI): 0.23–0.42) in moderate renal impairment, not reported in mild renal impairment, and HR: 0.55 (95% CI: 0.46–0.65) for nonrenal impairment patients. Comparing edoxaban 60 mg against warfarin resulted in HR: 0.63 (95% CI: 0.50–0.81) in moderate renal impairment, not reported in mild renal impairment, and HR: 0.88 (95% CI: 0.76–1.03) in nonrenal impairment. In patients with CrCl 25–49 mL/min, indirect comparison between DOACs showed less major bleeding with apixaban compared to dabigatran, rivaroxaban, and edoxaban 60 mg, but not edoxaban 30 mg. Edoxaban 30 mg demonstrated less major bleeding in all comparisons to other DOACs. The HR of major bleeding for edoxaban 30 mg versus edoxaban 60 mg was estimated as 0.49 (95% CI: 0.33–0.72). In patients with CrCl 50–79 mL/min, edoxaban 30 mg offered a lower major bleeding profile compared to other DOACs. Edoxaban 30 mg versus edoxaban 60 mg showed HR of bleeding as 0.63 (95% CI: 0.50–0.79) [45]. The PK differences such as *T*_max_, *T*_1/2_, *F*, and various degrees of renal elimination could potentially confound the results of this metaregression. A recent multicenter, open-label, 3-parallel-group, phase 3 study in Japanese patients undergoing lower limb replacement has evaluated the safety of edoxaban administered for 11 to 14 days. Patients with mild renal insufficiency or CrCl ≥ 50 to ≤ 80 mL/min received oral edoxaban 30 mg once daily. Patients with severe renal insufficiency or CrCl > 20 to < 30 mL/min were randomized to receive edoxaban 15 mg once daily or fondaparinux 1.5 mg daily. Patients with severe renal insufficiency or CrCl ≥ 15 to ≤ 20 mL/min received edoxaban 15 mg daily. Edoxaban was given 12–24 hours after surgery. Patients undergoing hemodialysis, high risk of bleeding, risk of thromboembolism, hepatic dysfunction, spinal anesthesia, inability to take oral medication, and abnormal bleeding after surgery were excluded. There was no major bleeding in any groups; and clinically relevant bleeding occurred at a rate of 6.7% in mild renal insufficiency (CrCl ≥ 50 to ≤ 80 mL/min on edoxaban 30 mg), 3.4% in severe renal insufficiency (CrCl ≥ 15 to < 30 mL/min on edoxaban 15 mg), 0% in patients in severe renal insufficiency on edoxaban 15 mg, 4.5% in severe renal insufficiency (CrCl ≥ 20 to ≤ 30 mL/min on edoxaban 15 mg), and 5% in severe renal insufficiency (CrCl ≥ 20 to < 30 mL/min on fondaparinux 1.5 mg). There was no VTE reported in any treatment groups. This study demonstrated that edoxaban 15 mg is safe in patients with severe renal impairment [46]. The limited number of patients in a subpopulation of Asian patients makes the applicability of data restrictive. The manufacturer recommends dose reduction by 50% in patients with CrCl 15–50 mL/min [17].

## 4. Edoxaban in Venous Thromboembolism (VTE) Trials

### 4.1. Primary Thromboprophylaxis after Knee Surgery

The incidence of 42%–57% for deep vein thrombosis (DVT) on screening, and 0.1%–2.0% for pulmonary embolism (PE), without the use of pharmacologic prophylaxis within 2 weeks in patients undergoing total hip arthroplasty (THA) has been described. In total knee arthroplasty (TKA) setting, incidence of DVT climbs to 41%–85% and prevalence of PE could be as high as 0.1%–1.7%. In hip fracture surgery (HFS), the incidence of DVT is estimated at 40%–60% and prevalence for PE at 0.3%–7.5% [47, 48]. In patients undergoing major orthopedic surgery (TKA or THA), the American College of Chest Physicians (ACCP) recommends utilizing one of the following pharmacological antithrombotics rather than no antithrombotic prophylaxis: LMWH, fondaparinux, dabigatran, apixaban, rivaroxaban, low-dose unfractionated heparin (LDUFH), adjusted-dose VKA, aspirin, or an intermittent pneumatic compression device (IPCD) for a minimum of 10 to 14 days. For HFS, the ACCP guidelines do not recommend utilizing DOACs for thromboprophylaxis. These guidelines suggest the use of LMWH in preference to the other agents and adding an IPCD during the hospital stay and suggest extending thromboprophylaxis for up to 35 days. In patients who decline injections, guidelines recommend using apixaban or dabigatran [49]. In the setting of TKA, a dose ranging study of edoxaban (5 mg, 15 mg, 30 mg, and 60 mg) once daily (first dose 6–24 hours after surgery) versus placebo in 523 patients has been conducted in a randomized, double-blind, placebo-controlled fashion for 11–14 days. Importantly, patients at high risk for bleeding, that is, history of intracranial bleeding, gastrointestinal bleeding, intraocular bleeding, peptic ulcer disease, elevated prothrombin time (PT), or activated partial thromboplastin time (aPTT) above the upper normal limit (UNL), hemoglobin (Hgb) < 9 g/dL or platelets of < 100,000/mm^3^, systolic blood pressure (SBP) > 160 mmHg or diastolic blood pressure (DBP) > 100 mmHg, inherited coagulation abnormality, history of VTE, myocardial infarction, cerebral infarction, or transient ischemic attack (TIA), body weight < 40 kg, concurrent antiplatelet therapy, pregnancy, and clinically significant hepatic dysfunction, that is, liver enzymes (LFTs) or total bilirubin above 1.5 times upper limit of normal (UNL). The incidence of thromboembolic complications (DVT, PE) or primary endpoint in placebo and edoxaban (5 mg, 15 mg, 30 mg, 60 mg) was 48.3%, 29.5%, 26.1%, 12.5%, and 9.1%, respectively, *P* < 0.001, for all comparisons (placebo versus edoxaban). No major bleeding was observed in placebo and in only 1/106 (0.9%) in edoxaban 60 mg daily, all statistically nonsignificant. The clinically relevant nonmajor bleeding (CRNM) was 3.9% in placebo, 1.9% in edoxaban 5 mg, 3.8% in edoxaban 15 mg, 3.9% in edoxaban 30 mg, and 3.8% in edoxaban 60 mg groups accordingly. Notably, the incidence of treatment-related bleeding increased with higher doses of edoxaban (*P* = 0.004). There were no elevations in liver enzymes associated with edoxaban therapy. Additionally, single doses of edoxaban (5–60 mg) resulted in dose-dependent increases in anti-FXa activity, demonstrating target-specific effect versus placebo [50]. Nonpharmacological thromboprophylaxis (21–28% IPC and 72% elastic stockings) was used in addition to edoxaban, but no stratified analysis was provided on their effect on the outcomes. The effect of anti-FXa on clinical outcomes was not measured. This study confirmed the higher degree of bleeding with higher doses of edoxaban. A retrospective study of 300 patients undergoing TKA analyzed the safety and efficacy of edoxaban 15 mg daily for the prevention of DVT administered for 14 days. This study compared edoxaban 15 mg daily versus enoxaparin 20 mg twice daily versus fondaparinux 1.5 mg daily. The incidence of total DVT in enoxaparin, fondaparinux, and edoxaban was 28%, 28%, and 22% accordingly, not statistically significant, and no PE was noted. Hemoglobin levels were lower in patients with edoxaban than in patients with enoxaparin and fondaparinux on postoperative days; however, the difference was not statistically significant. Finally, the incidence of hepatic dysfunction was lower in patients with edoxaban than in patients with enoxaparin and fondaparinux [51]. Applying the results of a retrospective study into clinical practice poses many challenges. This study provided very limited information of patient's baseline characteristics, that is, renal function, concomitant use of IPCS, and comorbidities. Utilization of different doses of fondaparinux (1.5 mg) and enoxaparin (20 mg twice daily) is questionable in western countries as such doses are not recommended. Finally, no conclusion can be drawn for the efficacy and safety of edoxaban 30 mg daily. In a randomized double-blind, double-dummy trial of TKA patients (*n* = 716), the safety and efficacy of edoxaban 30 mg (first dose given 6–24 hours after surgery) were compared to enoxaparin 20 mg twice daily (first dose 6–24 hour after surgery) for 11–14 days. Patients at high risk for bleeding, high risk for VTE, body weight < 40 Kg, CrCl < 30 mL/min, hepatic dysfunction, concomitant antiplatelet, or thrombolytic agents or pregnant patients were excluded. Patients in whom epidural catheter could not be removed by 2 hours prior to the initiation of study were not enrolled as well. The primary efficacy outcome (thromboembolic events) occurred in 7.4% and 13.9% patients in the edoxaban and enoxaparin groups, respectively (relative risk reduction = 46.8%), demonstrating noninferiority (*P* < 0.001) and superiority (*P* = 0.010) of edoxaban over enoxaparin. In the edoxaban and enoxaparin groups, the major bleeding (primary safety outcome) occurred in 1.1% versus 0.3% of patients (*P* = 0.373). Major or CRNM bleeding occurred in 6.2% versus 3.7% patients (*P* = 0.129), accordingly. There was no report of drug-induced liver injury. It is important to note that in each group approximately 22% were permitted to use IPCD and 62% elastic stockings as nonpharmacological prophylaxis measures [32]. Evidence has shown that addition of IPCD to pharmacological methods further decreased the incidence of VTE in orthopedic surgical patients; however, no analysis was conducted to examine the effect of covariates such as use of IPCD. Together, all of edoxaban data in TKA trials may not be applicable to patients in western countries as weight-based dosing of pharmacological thromboprophylaxis and methods of diagnosis may differ.

### 4.2. Primary Thromboprophylaxis after Hip Surgery

Edoxaban has been studied in total hip replacement (THR) trial for prevention of VTE. A randomized double-blind dose response of edoxaban on 903 patients undergoing THR has been conducted. In this trial, THR patients were randomized to edoxaban (15 mg, 30 mg, 60 mg, and 90 mg) versus dalteparin (2,500 units initially then 5,000 units) daily thereafter for 7 days. Patients were excluded if they have known or suspected bleeding or coagulation disorder, hemorrhagic stroke, nonhemorrhagic stroke within the past three months, intraocular hemorrhage, intracranial malignancy, gastrointestinal bleeding, or documented peptic ulcer within the past three months, if they are expected to receive epidural catheter, if they have received spinal or epidural anesthesia with traumatic tap, if they have abnormal PT and aPTT time, positive serology for hepatitis A, B, and C, known drug or alcohol dependence within the past 12 months, estimated survival of less than 12 months, liver function tests greater than 1.5 times UNL, systolic and diastolic blood pressure > 180/110 mmHg, Hgb < 9 g/dL, platelet count < 100,000/mm^3^, if they receive aspirin > 100 mg per day or required concomitant clopidogrel or dipyridamole or nonsteroidal anti-inflammatory drugs, and if they received therapeutic dose of VKA or fibrinolytics within the past 10 days. Primary efficacy result, incidence of venographically proven VTE, was reported as 43.5% (dalteparin), 28.2% ((edoxaban 15 mg) versus dalteparin *P* = 0.005), 21.2% ((edoxaban 30 mg) versus dalteparin; *P* < 0.001), 15.2% ((edoxaban 60 mg) versus dalteparin; *P* < 0.001), and 10.6% ((edoxaban 90 mg) versus dalteparin; *P* < 0.001). Primary safety results, incidence of all bleeding, were reported as 0.6% (dalteparin), 2.1% (edoxaban 15 mg versus dalteparin; *P* = 0.250), 1.8% (edoxaban 30 mg versus dalteparin; *P* = 0.122), 4.9% (edoxaban 60 mg versus dalteparin), and 4.0% (edoxaban 90 mg versus dalteparin). There was no report of edoxaban induced liver injury. This study showed that edoxaban was effective in reduction of VTE in patients undergoing THR. This study was conducted in a patient population that had a different population PK than patients in North America, and generalizability is limited. No information was provided on the addition of nonpharmacological thromboprophylaxis [33]. A population PK based study of 1,795 patients enrolled in 10 phase 1 studies of edoxaban has been published for patients undergoing THR. Range of plasma concentration of edoxaban (15 mg, 30 mg, 60 mg, and 90 mg) was reported as *C*_max,ss_ (200–300 ng/mL), AUC_ss_ (2000–3000 ng*∗*h/mL), and *C*_min,ss_ (20–50 ng/mL) and there was a significant predictor of decreasing incidence of VTE in patients undergoing THR: *P* < 0.005 for all variables; and no relationship was identified between edoxaban exposure and bleeding [34]. The applicability of these pharmacometric analyses might be to guide the future clinical trials in dosing and monitoring edoxaban in patients undergoing orthopedic surgery. Edoxaban 15 or 30 mg daily versus enoxaparin 20 mg twice daily has been studied for the prevention of VTE in patients undergoing THA in a randomized, active controlled, double-blind, phase II trial of 264 patients. Patients who required revision of THA and had history of intracranial bleeding, history of intraocular bleeding, intracranial tumor, gastrointestinal bleeding or peptic ulcer disease within the past 90 days, or history of symptomatic VTE, patients weighing < 40 kg and using antithrombotics, CrCl < 30 mL/min, and patients with evidence of hepatic dysfunction (LFTs > 2 times UNL and total bilirubin > 1.5 times UNL) were excluded. Edoxaban was started 6–24 hours after surgery and enoxaparin 24–36 hours after surgery for 11–14 days at which patients underwent venography. The primary endpoint (composite of asymptomatic VTE or symptomatic DVT) occurred in 3.8% of edoxaban 15 mg, 2.8% in edoxaban 30 mg group (incidence difference of 1.1%), and 4.1% in enoxaparin group. The incidence difference was −0.2% and −1.3% in edoxaban 15 mg and 30 mg versus enoxaparin, respectively (*P* = 1.000). There were no VTE-related deaths. The incidence of overall bleeding was 18% in edoxaban 15 mg, 21.2% in edoxaban 30 mg, and 21.8% in enoxaparin groups (*P* = 1.000). This study demonstrated that edoxaban once daily showed similar efficacy while maintaining safety as enoxaparin for the prevention of VTE after THA. Approximately 50% of patients were allowed to have IPCD therapy for sole foot, 40% IPCD for lower legs and thigh, and 80% for elastic stockings [52]. The dose of enoxaparin for prophylaxis was different than the dose recommended in the western countries; however, enoxaparin 20 mg twice daily might be appropriate for smaller frame patients. Currently, there are no large scale trials in America and Europe to compare the different doses of edoxaban and enoxaparin. Edoxaban 30 mg daily versus enoxaparin 20 mg twice daily has also been studied for the prevention of VTE in patients undergoing THA in a randomized, double-blind, double-dummy, noninferiority phase III trial involving 610 patients. Edoxaban was started 6–24 hours after surgery and enoxaparin was initiated 24–36 hours after surgery for 11 to 14 days. VTE events occurred in 6.9% of enoxaparin group and 2.4% of edoxaban group (*P* < 0.001 for noninferiority). No symptomatic DVT or PE was noted in both treatment groups. The incidence of major and CRNM bleeding events was 3.7% versus 2.6% in the enoxaparin and edoxaban groups, respectively (*P* = 0.475). The major bleeding rates were 2.0% versus 0.7% in the enoxaparin and edoxaban groups, respectively. This study was presented as an abstract and the full paper has not been published yet [35].

### 4.3. Primary Thromboprophylaxis after Hip Fracture Surgery (HFS)

To date, one multicenter, open-label, active comparator, phase 3 trial of 92 patients has compared edoxaban 30 mg to enoxaparin 20 mg twice daily for VTE prevention after HFS in Japanese patients for 11–14 days. First dose of edoxaban was given 6–24 hours and enoxaparin 24–36 hours after surgery. Patients were excluded if they were at increased risk of bleeding (e.g., history of intracranial bleeding and recent gastrointestinal bleeding) and patients with prior VTE, recent myocardial infarction, cerebral infarction, transient ischemic attack, body weight < 40 Kg, use of concomitant antithrombotics, CrCl < 30 mL/min, evidence of hepatic impairment, or abnormal bleeding at the site of anesthesia were also excluded. The primary endpoint of major or CRNM bleeding occurred in 3.4% (95% CI: 0.9–11.5) of edoxaban and 6.9% (95% CI: 1.9–22.0) of enoxaparin, for absolute difference of −3.5% (95% CI: −18.8–6.0). The secondary endpoint of composite VTE occurred in 6.5% of edoxaban (95% CI: 2.2–17.5) and 3.7% of enoxaparin (95% CI: 0.7–18.3), with absolute difference of 2.8% (95% CI: −12.4–14.2). There were no major adverse events such as death or liver toxicity related to treatment. This study confirmed the efficacy and safety of edoxaban for VTE prevention in high-risk postorthopedic surgical patients [36]. Patient eligibility and recruitment variations along with dosing differences for VTE prevention might impede the generalizability of these findings. Edoxaban is not indicated for the prevention of VTE postorthopedic surgery [17].

### 4.4. Primary Thromboprophylaxis in Acutely Ill Medical Patients

Hospitalized patients admitted for acute medical illnesses such as infection, advanced age, congestive heart failure, acute exacerbation of chronic obstructive lung disease, acute rheumatological disease, immobilization, cancer, respiratory failure, and prior history of thromboembolism are at risk for VTE [53]. Studies have shown the incidence of VTE in acutely ill hospitalized medical patients varies from 5% to 15% and this risk could be reduced by 50% to 75% with appropriate pharmacological thromboprophylaxis [54–56]. The International Medical Prevention Registry on Venous Thromboembolism (IMPROVE) registry (*n* = 15, 156) showed suboptimal VTE prophylaxis rates, less than 50% [57]. The ACCP recommends pharmacological thromboprophylaxis with LMWH, LDUFH, or fondaparinux for acutely ill hospitalized medical patients at increased risk of thrombosis [58]. Currently, there are no recommendations for thromboprophylaxis with OFXaIs in medically ill hospitalized patients. Although data exists for apixaban and rivaroxaban thromboprophylaxis in this setting, to date, no published data exists for edoxaban prophylaxis in acutely ill medical patients [59, 60]. Generalizability of these data to edoxaban is not clinically advisable, for there are clear PK differences among OFXIs such as protein binding, renal clearance, hepatobiliary elimination, and prophylaxis dosing differences. Edoxaban is not indicated for the prevention of VTE in acutely ill hospitalized patients.

### 4.5. Initial Treatment of VTE

The overall annual incidence of VTE (DVT and PE) is estimated between 1 and 2 per 1000 of population or between 300,000 and 600,000 cases per year with average treatment cost of $7594 to $16,664 per case and 10 to 30% of all patients suffer mortality [61]. The ACCP recommendation for the VTE treatment consists of administration of either UFH or LMWH or fondaparinux for a minimum of 3 months for the first VTE; selected patients may be transitioned to warfarin [62]. OFXaIs have been studied for the initial treatment of acute VTE in several large randomized, noninferiority trials. Rivaroxaban and apixaban are considered noninferior to the standard-of-care VTE treatment in the general population [63–65]. The Hokusai-VTE investigated the role of edoxaban versus warfarin for the treatment of symptomatic VTE (DVT and PE) in a randomized, double-blind, double-dummy, noninferiority design trial. All patients were initially treated with heparins (UFH or enoxaparin) for a minimum of 5 days followed by warfarin (target INR 2-3) or edoxaban 60 mg daily unless the body weight was < 60 Kg or CrCl was 30–50 mL/min or were on potent P-glycoprotein inhibitor, in which cases edoxaban 30 mg daily was given for a minimum of 3 months in all patients and for a maximum of 12 months. The following patients were excluded: patients who required thrombectomy, insertion of a caval filter, or use of fibrinolytic agent; patients with CrCl < 30 mL/min, significant liver disease (e.g., acute hepatitis, chronic active hepatitis, positive hepatitis B antigen or hepatitis C antibody, and cirrhosis) or alanine transaminase (ALT) > 2 times UNL, total bilirubin 1.5 times UNL, active bleeding or high risk for bleeding, SBP > 170 mmHg, or DBP > 100 mmHg; pregnant patients; patients with chronic condition requiring treatment with nonsteroidal anti-inflammatory drugs (NSAIDs) including both cyclooxygenase-1 (COX-1) and cyclooxygenase-2 (COX-2) inhibitors for 4 days/week; patients requiring treatment with aspirin in a dosage of > 100 mg/per day or dual antiplatelet therapy (any two antiplatelet agents including aspirin plus any other oral or intravenous (IV) antiplatelet drug); patients requiring treatment with the potent P-gp inhibitors ritonavir, nelfinavir, indinavir, or saquinavir; however, systemic use of the strong P-gp inhibitors erythromycin, azithromycin, clarithromycin, ketoconazole, or itraconazole at the time of randomization was permitted. The primary efficacy outcome (first recurrent VTE or VTE-related death) occurred in 3.2% in edoxaban and 3.5% in warfarin groups, HR: 0.89 (95% CI: 0.70–1.33; *P* < 0.01 for noninferiority). The primary safety outcome (first major or CRNM bleeding) occurred in 8.5% of edoxaban and 10.3% of warfarin groups, HR: 0.81 (95% CI: 0.71–0.94; *P* = 0.004 for superiority). Therapeutic INR range occurred in 65% of patients and adherence to edoxaban was 80%. Approximately 12% of patients in each arm were treated for 3 months, 26% for 3 to 6 months, 62% for greater than 6 months, and 40% for 12 months. In patients with PE, the rates of recurrent VTE occurred in 3.3% in the edoxaban group and 6.2% in the warfarin group, HR: 0.52 (95% CI: 0.28–0.98) and for those with documented right ventricular dysfunction based on computed tomography the HR of recurrent VTE was 0.42 (95% CI: 0.15–1.20) in edoxaban versus warfarin. Compared to warfarin, the rate of recurrent VTE in patients on edoxaban 30 mg was 3.0% versus 4.2%, HR: 0.73 (95% CI: 0.42–1.26). The rates of arterial thromboembolism in both arms were less than 0.6% and rates of liver injury were less than 2% in each arm [37]. The Hokusai-VTE showed noninferiority of edoxaban; however, clinicians should be aware that edoxaban was started after the initial treatment with heparins (5 days) as some patient with life-threatening VTE may require immediate procedures such as thrombectomy with or without thrombolysis. There is no subgroup analysis on edoxaban 60 mg and 30 mg to determine the dose effect on safety and efficacy outcomes. Edoxaban 60 mg orally daily is the approved dose for VTE treatment, unless CrCl is between 15 and 50 mL/min or body weight < 60 kg or patient was on concomitant Pap inhibitor in which 30 mg orally daily should be used (Table 1) [17].

### 4.6. Extended Treatment of VTE

The rate of VTE recurrence after discontinuation of therapy has been reported as 11.0% after 1 year, 19.6% after 3 years, and 29.1% after 5 years [66]. The risk of mortality is estimated as 3.6% for a recurrent VTE and 11.3% for a major bleed [67]. The ACCP guidelines recommend chronic anticoagulation for patients with recurrent VTE [61]. Although data is available for extended treatment for VTE with apixaban or rivaroxaban [68, 69], limited data exists for edoxaban.

### 4.7. Primary Thromboprophylaxis in Ambulatory Cancer Patients Receiving Chemotherapy

The association of malignancy and VTE is well described [70]. Pancreatic, brain, gastric, esophageal, and renal cell cancers and acute myelogenous leukemia confer a higher cumulative risk of VTE than breast, prostate, and melanoma malignancies [71]. In a population-based cohort of cancer patients with VTE, the cumulative incidence of VTE recurrence was 26.7 to 52.2% from the incidence of first VTE with mortality of 55.9% to 85.2% over 10 years [72]. In a study of cancer patients (*n* = 3,805) with VTE, risk of bleeding was 4.1% with 29% mortality. Predictive variables of bleeding were CrCl ≤ 30 mL/min, immobility ≥ 4 days, history of recent major bleeding, and metastatic cancer [73]. Predictive variables such as site of cancer, platelet count > 350,000/mm^3^, hemoglobin < 10 g/dL, use of erythropoiesis-stimulating agents, leukocyte count > 11 × 10^9^/L, and body mass index ≥ 35 kg/m^2^ may assist in identifying high-risk cancer patients for developing VTE [74]. Although randomized phase II study (tolerability trial) data exists for apixaban in this setting [75], no published data exists for edoxaban.

### 4.8. Initial Treatment of VTE in Cancer Patients

Enrolling cancer patients in clinical trials of DOACs has posed a challenge for investigators, due to underlying coagulopathy and thrombocytopenia and uncertain prognosis. VTE treatment trials of rivaroxaban enrolled 5 to 7% active cancer patients (acute DVT and continued treatment), 2.5% apixaban patients with active cancer, and 9% edoxaban patients [37, 63–65]. A subgroup analysis of patients enrolled in EINSTEIN-DVT and EINSTEIN-PE with active cancer (diagnosed at baseline or during treatment) has been published. Overall rivaroxaban was deemed similar in efficacy and safety to VKA in a subgroup of cancer patients [76]. Subgroup analysis may create false positive results by decreasing power, alteration of hypothesis, and generation of a mere observation. None of OFXaIs VTE treatment trials were designed to look at cancer patients as a subgroup. A systemic review of 4 randomized controlled phase III trials of 19,060 of which 759 with cancer randomized to either DOAC or SOC demonstrated efficacy (OR: 0.56 (95% CI: 0.28 to 1.13)) in DOACs versus SOC group. Safety outcomes comparing DOAC to VKA of yielded OR: 0.88 (95% CI: 0.57 to 1.35) [77]. A recent meta-analysis of six studies (two with dabigatran, two with rivaroxaban, one with edoxaban, and one with apixaban) comparing DOACs to SOC for treatment of VTE including patients with cancer demonstrated that VTE recurred in 3.9% (23/595) and 6% (32/537) of patients with cancer treated with DOACs and SOC, respectively (OR: 0.63 (95% CI: 0.37–1.10; *I* ^2^ 0%)). Major bleeding occurred in 3.2% and 4.2% of patients receiving DOACs and SOC, respectively (OR: 0.77 (95% CI: 0.41–1.44; *I* ^2^ 0%)) [78]. This meta-analysis, despite a high degree of homogeneity (*I* ^2^ = 0%), analyzed dabigatran (DTI) in addition to OFXaIs. Dabigatran weight in this meta-analysis was 37% which might have skewed the results. Dabigatran PD/PK properties are completely different and should not be compared to OFXaI in the absence of direct comparison randomized head-to-head clinical trial in VTE setting. Currently, the American Society of Clinical Oncology (ASCO), National Comprehensive Cancer Network (NCCN), and ACCP recommend LMWH as the first line and warfarin as the second line for treatment of VTE in cancer patients. These guidelines currently do not recommend DOACs in cancer patients due to lack of randomized clinical trials [79, 80]. Another point to consider is that none of the trials with DOACs were set to analyze cancer subset data analysis. A phase III study comparing low-molecular-weight heparin versus edoxaban for the treatment of cancer-associated VTE is underway.

## 5. Edoxaban Studies in Acute Coronary Syndrome (ACS)

OFXaIs have been investigated in patients with recent ACS. In the ATLAS-ACS 2-TIMI 51 trial, rivaroxaban reduced the rates of death from cardiovascular causes (2.7% versus 4.1%, *P* = 0.002) and, compared with placebo, rivaroxaban increased the rates of major bleeding not related to coronary artery bypass grafting (2.1% versus 0.6%, *P* < 0.001) and intracranial hemorrhage (0.6% versus 0.2%, *P* = 0.009), without a significant increase in fatal bleeding (0.3% versus 0.2%, *P* = 0.66) [81]. In the APRAISE-2, addition of apixaban to antiplatelet therapy did not reduce the risk of cardiovascular death, myocardial infarction, or ischemic stroke versus placebo, 13.2 versus 14.0%, *P* = 0.51, but significantly increased the risk of major bleeding (2.4 versus 0.9%, *P* = 0.001) [82]. To our knowledge, there is no clinical trial published for the role of edoxaban in patients with ACS.

## 6. Prevention of Stroke in Nonvalvular Atrial Fibrillation (NVAF)

A three-arm, randomized, double-blind, double-dummy trial (ENGAGE AF-TIMI 48) compared once daily edoxaban (30 mg and 60 mg) with warfarin in 21,105 patients with NVAF. Patients with an estimated CrCl < 30 mL/min, high risk of bleeding, use of dual antiplatelet therapy, moderate to severe mitral stenosis, acute coronary syndromes, stroke within 30 days before randomization, and an inability to adhere to study procedures were excluded. The annual rate of the stroke or systemic embolism during treatment was 1.50% with warfarin, as compared with 1.18% with high-dose edoxaban (HR: 0.79 (97.5% CI: 0.63–0.99); *P* < 0.001 for noninferiority) and 1.61% with low-dose edoxaban (HR: 1.07 (97.5% CI, 0.87–1.31); *P* = 0.005 for noninferiority). In the intention-to-treat analysis, there was a trend favoring edoxaban 60 mg versus warfarin (HR: 0.87 (97.5% CI: 0.73–1.04); *P* = 0.08) and an unfavorable trend with edoxaban 30 mg versus warfarin (HR: 1.13 (97.5% CI: 0.96–1.34); *P* = 0.10). The annual rate of major bleeding was 3.43% with warfarin versus 2.75% with 60 mg edoxaban (HR: 0.80 (95% CI: 0.71–0.91); *P* < 0.001) and 1.61% with 30 mg edoxaban (HR: 0.47 (95% CI: 0.41–0.55); *P* < 0.001). The corresponding annual rates of death from cardiovascular causes were 3.17% versus 2.74% (HR: 0.86 (95% CI: 0.77–0.97); *P* = 0.01) and 2.71% (HR: 0.85 (95% CI: 0.76–0.96); *P* = 0.008), and the corresponding rates of the key secondary endpoint (a composite of stroke, systemic embolism, or death from cardiovascular causes) were 4.43% versus 3.85% (HR: 0.87 (95% CI: 0.78–0.96); *P* = 0.005) and 4.23% (HR: 0.95 (95% CI: 0.86–1.05); *P* = 0.32). Warfarin was within the therapeutic range in 58% of patients. This study showed noninferiority of edoxaban to SOC and significantly lower bleeding rates in prevention of stroke in patients with NVAF [38]. The subgroup analysis of patients with CrCl > 95 mL/min in ENGAGE AF-TIMI 48 showed higher rates of stroke and systemic embolism events (SEE) in edoxaban versus warfarin 1.0 versus 0.6 (HR: 1.87 (95% CI: 1.10–3.17)). The rate of ischemic stroke was higher relative to warfarin in the patients with CrCl > 95 mL/min (HR: 2.16 (95% CI: 1.17, 3.97)). The PK data indicated that patients with CrCl > 95 mL/min had lower plasma edoxaban levels, along with a lower rate of bleeding relative to warfarin than patients with CrCl ≤ 95 mL/min. Consequently, edoxaban should not be used in patients with CrCl > 95 mL/min [17]. The dose of edoxaban for NVAF is 60 mg orally daily unless CrCl is 15–50 mL/min in which the dose should be decreased to 30 mg (Table 1) [17].

## 7. Thrombolysis Management in Edoxaban-Treated Patients Who Develop Acute Ischemic Stroke (AIS)

Despite edoxaban prophylaxis in NVAF, some patients develop AIS. Intravenous administration of recombinant tissue plasminogen activator (rTPA) is the only FDA-approved therapy for treatment of patients with AIS. The American Heart Association and the American Stroke Association recommend thrombolysis with rTPA in AIS patients with 3–4.5 hours of onset of stroke symptoms. Data on thrombolysis in edoxaban-treated patients with AIS are very limited. Patients who are already on edoxaban pose many challenges because of increased risk of major bleeding complications when the rTPA is concurrently administered. Unless rapidly performed PT, aPTT, and appropriate direct factor Xa activity assays are normal or the patient has not received a dose for > 2 days (assuming normal renal function), AHA/ASA guidelines do not recommend thrombolysis. Unfortunately, most of the tests are time-consuming to meet the 3–4.5-hour thrombolysis window [83].

## 8. Measurement of the Anticoagulant Effect of Edoxaban

The effect of edoxaban levels on coagulation parameters such as PT (prothrombin time), INR (international normalized ratio), aPTT (activated partial thromboplastin time), and factor Xa has not been extensively studied. An ex vivo study of 12 healthy volunteers assessed the antithrombotic effect of edoxaban 60 mg on coagulation parameters. Thrombin generation decreased by 28% and 10% at 1.5 hours and 10 hours. Changes in PT, INR, and anti-FXa activity correlated well with plasma drug concentrations (*R* ^2^ = 0.79, 0.78, and 0.85), but aPTT changes did not (*R* ^2^ = 0.40). Drug levels at 1.5, 5, and 12 hours after edoxaban 60 mg were 240 ± 16, 127 ± 6, and 37 ± 3 ng/mL. The effect of drug level on the thrombus size reduction was not measured [84]. Edoxaban concentration in plasma after multiple dose administration has been evaluated. Edoxaban 90 mg daily provided *C*_max/min_ for day 1 as 451/10.2 (ng/mL) and 424/16.4 (ng/mL) on day 10. Edoxaban 60 mg twice daily achieved *C*_max/min_ of 347/38.3 (ng/mL) on day 1 and 397/80.3 (ng/mL) on day 10. Finally, edoxaban 120 mg daily showed *C*_max/min_ of 387/11.1 (ng/mL) on day 1 and 406/15.6 (ng/mL) on day 10 [18]. A phase 2 study on edoxaban for prevention of stroke in NVAF measured the *C*_max/min_ of edoxaban in 1,146 patients. At steady state, the median *C*_max/min_ was 80/10 (ng/mL) for edoxaban 10 mg daily, 175/20 (ng/mL) for edoxaban 60 mg daily, 120/40 (ng/mL) for edoxaban 30 mg twice daily, and 225/75 (ng/mL) for edoxaban 60 mg twice daily. This study showed higher bleeding rates with higher total daily doses of edoxaban. Interestingly, *C*_min_ correlated more closely with bleeding; that is, frequency of bleeding was higher with edoxaban 30 mg twice daily versus edoxaban 60 mg daily. No analysis of edoxaban level on primary outcome such as stroke was evaluated [85]. A recent study has shown the significant variation is prolonged in PT and aPTT postedoxaban dose diluted to reach plasma concentrations of 50–400 ng/mL. The PT prolongation remained variable and dependent on specific reagent, but prolonged aPTT variability remained smaller; however, thrombin generation assay proved to be sensitive [86]. Differences in PK variables (*T*_max_, *C*_max_, and *C*_min_) in edoxaban studies have been observed. There is no consensus at this point to make recommendations for monitoring edoxaban in special populations (obesity, underweight, and renal insufficiency). Lack of a standardized assay to monitor FXa poses a dilemma for clinicians assessing safety and efficacy of treatment with edoxaban.

## 9. Management of Bleeding Complications

Bleeding is a complication of anticoagulation therapy. Availability of antidote can prevent potential unfavorable outcomes associated with hemorrhagic events. Rates of major bleeding in edoxaban group versus warfarin group have been reported as 1.4% versus 1.6% in a VTE treatment trial [37]. In the setting of NVAF, the rates of major bleeding with high-dose edoxaban (60 mg daily) group were 2.75% per year versus 1.61% per year in low-dose edoxaban (30 mg daily) (Table 5) [38]. To date, there are not any clinical trials investigating the reversal of edoxaban in humans. In an edoxaban-anticoagulated animal study, the prothrombin complex concentrate (PCC), recombinant factor VIIa (rFVIIa), and activated prothrombin complex concentrate (aPCC) shortened PT prolonged by edoxaban. Among those, rFVIIa and aPCC showed potent activities in reversing the PT prolongation by edoxaban. rFVIIa (1 and 3 mg/kg) and aPCC (100 U/kg) significantly reversed edoxaban (1 mg/kg/h) induced prolongation of bleeding time. In venous thrombosis model, no potentiation of thrombus formation was observed when the highest dose (3 mg/kg) of rFVIIa was added to edoxaban (0.3 and 1 mg/kg/h) compared with the control. This study indicated that rFVIIa, aPCC, and PCC have the potential to be reversal agents for edoxaban [87]. In a rabbit model acute hemorrhage induced by edoxaban, blood loss was increased to 30 mL (2–44) and time to reach hemostasis was prolonged by 23 minutes (8.5–30) versus control group. Administration of 4F-PCC significantly reduced time to hemostasis to 8 minutes (6.5–14), *P* < 0.0001, versus control. In this study, PT (seconds) was 9.7 ± 0.9 in glucose saline 5% group versus 17.4 ± 1.7 in edoxaban 1,200 *μ*g/kg saline group, *P* < 0.0001. Addition of 4F-PCC (50 IU/kg) lowered the PT (s) to 13.5 ± 0.7, *P* < 0.0001. This study confirmed that 4F-PCC effectively normalizes PT [88]. Edoxaban (500 or 1,000 ng/mL) was added to blood sample from 6 healthy volunteers followed by rFVIIa (0.8 or 1.8 *μ*g/mL) or factor VIII inhibitor bypassing agent (FEIBA (0.75 or 1.5 U/mL)). In edoxaban-containing blood samples, reductions in measures of PT (*P* < 0.0001), aPTT (*P* < 0.0001), and anti-FXa (*P* < 0.0001) were observed when rFVIIa or aPCC was added. Intrinsic FXa activity was increased up to 20% and 31% of normal in the presence of edoxaban by rFVIIa and aPCC, accordingly. The onset of their impact on the anticoagulant effects of edoxaban was observed within 15 minutes and remained relatively unchanged. Results of this ex vivo study suggest that rFVIIa and aPCC rapidly reversed edoxaban-mediated anticoagulation effects based on PT and aPTT but had minimal effect based on intrinsic FX activity [89]. In acute overdose, if ingestion is within 3 hours, activated charcoal (50–100 gm) can be given along with fluid and red blood cell resuscitation [90]. Overall, the administration of 4-FPCC (50 units/kg) and aPCC (50 units/kg) and addition of rVIIa (90 *μ*g/kg) in refractory cases have been shown in animal studies to normalize thrombin generation, decrease PT, and reduce bleeding time. In a recent study of 110 healthy subjects (17 in part one and 93 in part two), the effect of 4-FPCC (50, 25, and 10 IU/kg) on edoxaban (60 and 180 mg) was evaluated in a double-blind, randomized, placebo, 2-way crossover design. Patients were excluded if they were on strong inhibitors or inducers of CYP3A4 or P-gp within the past 28 days, if they had recent major or minor bleeding, and if they had history of coagulopathy. The effects of 4-FPCC on bleeding duration (BD) and bleeding volume (BV) and PT were assessed. BD (minutes) and BV (mL) were completely reversed (above 100%) by 4-FPCC (50 IU/kg); however, PT did not (less than 50%). 4-FPCC (25 IU/kg) effect on BD, BV, and PT was suboptimal (less than 75%) and 4-FPCC (10 IU/kg) had no effect on BD, BV, and PT. No death or thrombotic events were reported. 4-FPCC demonstrated complete reversal effect of edoxaban on coagulation parameters [91, 92]. Currently, there are no specific antidotes for OFXaIs; however, 2 investigational antidotes are in phase 2 clinical trials. Andexanet alfa, a modified recombinant FXa, with terminal *T*_1/2_ of ~6 hours binds to OFXaIs specifically, thereby neutralizing its anticoagulant effects. Aripazine, a small synthetic molecule, universally targets OFXaIs, LMWHs, thrombin inhibitor (IIaI), and fondaparinux by binding them. Upcoming large scale trials should shed light onto these antidotes' safety and efficacy [93].

## 10. Drug-Drug Interactions with Edoxaban

Drug-drug or dietary supplement interactions with edoxaban may potentially predispose patients to bleeding or thromboembolism. Edoxaban's metabolism involves a major pathway via permeability glycoprotein (P-gp; ATP-binding cassette, subfamily B, membrane 1, or multidrug resistance-1 (MDR1)) transporter of the intestinal lining and a minor pathway of CYP3A4 [22]. Therefore, drugs that inhibit the P-gp pump can increase the level of edoxaban and drugs that induce the P-gp pump can lower the edoxaban level [23]. The addition of other antithrombotic or nonsteroidal anti-inflammatory agents to edoxaban may enhance the bleeding effect and combination should be avoided. Patients on edoxaban with recent coronary stent (on dual antiplatelet: aspirin and clopidogrel) may require lower doses of aspirin. Some inducers of P-gp transporter such as rifampin and St. John's Wort will lower the plasma concentration of edoxaban should be avoided. Cardiac medications such as verapamil and quinidine that are P-gp inhibitors require dose adjustment of edoxaban by 50%. Potent inhibitors of P-gp such as lansoprazole, omeprazole, azithromycin, erythromycin, ketoconazole, and itraconazole require 50% dose reduction of edoxaban if coadministered. Dose reduction of edoxaban is required if concomitant use of cardiac agents such as amiodarone or dronedarone (P-gp inhibitors) is warranted. Vascular endothelial growth factor (VEGF) inhibitors such as pazopanib, lapatinib, and sorafenib could potentially increase edoxaban plasma levels via P-gp inhibition. Dietary supplements such as vitamin E (>800 units per day) and fish oil can inhibit platelet aggregation and their combination with edoxaban may predispose patient to bleeding. There are supplements that have warfarin derivatives such as sweet clover, chamomile, and horseradish that should be avoided in combination with edoxaban, Table 2 [17, 24–29]. Patients on edoxaban should be counselled on drug or dietary supplement interactions.

## 11. Perioperative Management of Edoxaban Therapy

Clinicians should recognize that the data for use of edoxaban in perioperative setting is limited. Unless an emergent surgery is planned, elective surgery cases for patients on edoxaban require assessment of the risk of perioperative thromboembolism and the risk of bleeding. This assessment aids in determining the need for perioperative bridging with short-acting anticoagulants in the immediate perioperative setting. Patients with atrial fibrillation (CHADS_2_ (congestive heart failure, hypertension, age, diabetes, and stroke) score of 0 to 2, without prior history of stroke or transient ischemic attack (TIA)) and remote VTE event (history of more than 1 year in the past) are considered at low risk (less than 4% per year risk for ATE or less than 2% per month risk for VTE). This low-risk group does not require bridging. Patients with atrial fibrillation (CHADS_2_ score of 3 or 4), VTE (within the past 3 to 12 months), recurrent VTE, nonsevere thrombophilia, and active cancer disease are considered to be at intermediate risk (4 to 10% per year risk for ATE or 4 to 10% per month risk for VTE). This group may require bridging if bleeding risk is deemed low. Finally, patients with recent (<6 months) history of stroke or TIA, atrial fibrillation (CHADS_2_ score > 5, < 3 months history of stroke or TIA, and rheumatic valvular heart diseases), recent history of VTE ((history of < 3 months), severe thrombophilia, being positive for antiphospholipid antibodies, proteins C and S deficiency, and antithrombin deficiency) carry risk exceeding 10% per year for ATE and 10% per month for VTE. These patients require bridging with short-acting anticoagulants [30]. Once the patient was deemed an appropriate candidate for bridging, bleeding risk associated with procedure should be estimated as some procedures carry a higher risk of bleeding (major surgeries and renal biopsy) than simple dental extraction. For procedure with lower risk of bleeding, aiming for mild to moderate residual anticoagulant effect at surgery (12%–25%) or 2-3 half-lives should suffice, whereas for higher bleeding risk procedure, aiming for no or minimal residual anticoagulant effect (3%–6%) at surgery or 4-5 half-lives should suffice; therefore, edoxaban interruption can range from 24 to 72 hours (Table 3). If the patient is unable to tolerate oral tablets (prolonged nothing by mouth state) in the immediate postoperative period, transition LMWH or UFH infusion might be appropriate. Some institutions may not carry edoxaban due to formulary restrictions; in these situations, transition to other antithrombotics could be implemented [17, 31]. In order to assure a safe and effective practice, it is prudent to develop specific institutional guidelines or clinical practice algorithms for management of DOACs in the perioperative period by a multidisciplinary group compromising internists, surgeons, nursing staffs, and pharmacists.

## 12. Transition to Other Anticoagulants

Currently, there is no published trial evaluating the safety and efficacy role of bridging with edoxaban in the perioperative setting or transition to other antithrombotics. Transition to and from edoxaban requires estimation of CrCl and PK parameters such as *T*_1/2_ of each of the antithrombotics, monitoring INR and aPTT when necessary.  Table 4 will provide guidance to clinicians when bridging to and from edoxaban is required. Patients should be given a complete calendar and education of transition to and from edoxaban [17].

## 13. Current Approval Status

Edoxaban has been approved to reduce the risk of stroke or systemic embolism in patients with NVAF and for treatment of deep vein thrombosis and pulmonary embolism in USA, European Union, and Japan. Edoxaban has been approved for prophylaxis of deep vein thrombosis following orthopedic surgery in Japan. Edoxaban should not be used in patients with mechanical heart valves as no research trial has been conducted in this population. Dabigatran, an oral DTI, has been shown to be inferior to warfarin in reducing systemic embolism in this clinical setting [94].

## 14. Patient Satisfaction

Curtailing the need for frequent INR monitoring and administration of oral tables rather than injections could possibly improve patient compliance and satisfaction with anticoagulant therapy. Anti-Clot Treatment Scale (ACTS: burdens score range 12–60; benefits score range 3–15) and Treatment Satisfaction Questionnaire for Medication (TSQM: effectiveness; side effects; convenience, global satisfaction score range 0–100) are two frequently validated tools to assess the patient's satisfaction with anticoagulation and higher score equals higher satisfaction. In EINSTEIN-PE, mean ACTS for burdens score was 55.4 in rivaroxaban group and 51.9 in SOC group, *P* < 0.0001. The mean ACTS scores for benefits were 11.9 for rivaroxaban group and 11.4 for SOC group, *P* < 0.0001. TSQM scores for effectiveness, side effects, convenience, and global satisfaction were 73.3, 86.6, 81.6, and 80.7 for rivaroxaban, versus 69.6, 82.3, 71.8, and 73.0 for SOC group, *P* < 0.0001, for all groups [95]. In EINSTEIN-DVT significant benefits in terms of ACTS and TSQM scores were observed in rivaroxaban versus warfarin, *P* < 0.0001 [96]. One could presume the same satisfaction scores or even higher for edoxaban since it is dosed once daily. Future trials should provide additional evidence for benefits of edoxaban in terms of patient satisfaction.

## 15. Economic Impact

Recently, the cost-effectiveness of OFXaIs in orthopedic surgery patients has been investigated in a pharmacoeconomic decision model. In THR replacement model, the average cost per patient for LMWHs and that for oral FXa-Is were $18,897 and $18,762 accordingly; and quality-adjusted life-years (QALY) were 0.932 and 0.938. In TKR model, the average cost for LMWHs and that for oral FXa-Is were $18,891 and $18,804, respectively, with QALYs of 0.931 and 0.935 for LMWHs and OFXaIs, respectively. Overall sensitivity analysis indicated cost-effectiveness of OFXAIs is greater in 98% of patients undergoing major orthopedic surgery with the assumption of willingness-to-pay threshold of $50,000/QALY. Authors concluded that OFXaIs may be economically superior to LMWHs for VTE prevention in orthopedic surgery patients [97]. A recent pharmacoeconomic study examined the cost differences between DOACs and warfarin for treatment of atrial fibrillation and treatment of VTE in a hypothetical patient population. The incidence and prevalence of primary efficacy outcomes (ischemic stroke, hemorrhagic stroke, and systemic embolism), secondary efficacy outcomes (myocardial infarction, pulmonary embolism, and deep vein thrombosis), and safety endpoints (major bleeding and clinically relevant nonmajor bleeding) were extracted from all clinical trials of DOACs (dabigatran, rivaroxaban, apixaban, and edoxaban). Total medical cost difference measured as dollars/patient-years was −$204 for dabigatran versus warfarin, −$140 for rivaroxaban versus warfarin, −$495 for apixaban versus warfarin, and −$340 for edoxaban versus warfarin [98]. These results are promising; however, one must consider the acquisition cost differences across states and institutions and payer preference based on contracts.

## 16. Patient Education

Clinicians should become familiar with approved indications, pharmacodynamics, and pharmacokinetic properties of OFXaIs, duration of therapy, screen for drug interactions, communicate with other providers involved when an OFXaI is prescribed, and conduct a comprehensive patient education. Patient education should include signs and symptoms of bleeding, fall and injury precautions, reminders for the exact dose (color of the pill and frequency), and education on missed doses. Instructions on periodic monitoring of renal function, hemoglobin level, and platelet counts should be given to patients with significant comorbidities. There should be a plan of action in place, if pregnancy or planned procedures that require interruption of OFXaI occur.

## 17. Conclusion

Edoxaban seems safe and effective for prevention and treatment of VTE and prevention of stroke in NVAF. Edoxaban might be financially cost-effective and an attractive option compared to injections or warfarin. Edoxaban might provide better patient compliance and satisfaction with antithrombotic therapy. There is clearly lack of evidence for its role in cancer patients, perioperative bridging, transition to other anticoagulants, FXa monitoring, and reversibility for bleeding cases.

## Figures and Tables

**Table 1:** Edoxaban pharmacodynamics and pharmacokinetics [16–21].

Drug/mechanism of action	Edoxaban/direct oral factor Xa inhibitor (FXa-I) without antithrombin III

Indication and dosing guidelines	(1) Treatment of nonvalvular atrial fibrillation (NVAF) (i) 60 mg orally daily for CrCl greater than 50 to less than or equal to 95 mL/min (ii) 30 mg orally daily for CrCl 15–50 mL/min (iii) Do not use if CrCl is greater than 95 mL/min (Black Box Warning) (2) Treatment of venous thromboembolism (VTE) (i) 60 mg orally daily (ii) 30 mg orally daily if CrCl is 15–50 mL/min or body weight is less than 60 Kg or patient is on P-gp inhibitor

Protein binding/removed by dialysis	55%/No

*F* (%)	62% absorption in gastrointestinal tract Food does not affect the systemic exposure No data available for administration via feeding tube

*T*_max⁡_ (h)	1-2

Vd (L)	19.9

*T*_1/2_ (h)	10–14 with steady state reached in 72 hours

Metabolism	Minimal hepatic, undergoes biotransformation to various metabolites, the most abundant of which [M4] is formed through hydrolysis

Effect of P-gp/ABCG2 on metabolism	Minimal

Renal excretion (%)	50%

Biliary-intestinal excretion (%)	50%

Pregnancy category	C

Bioavailability, *F*; creatinine clearance, cytochrome P450 3A4 (CYP3A4/5), CrCl; half-life, *T*_1/2_; P-glycoprotein/ABCG2, P-gp/ABCG2; volume of distribution, Vd, time to reach maximum concentration in hours (h), *T*_max⁡_.

**(a):** 

Interacting drugs	Effect	Clinical Implications
Rivaroxaban, apixaban, dabigatran	Enhanced bleeding via additional antithrombotic effects	Avoid combination
Enoxaparin, dalteparin, tinzaparin, fondaparinux, Unfractionated Heparin, warfarin		Avoid combination
Streptokinase, alteplase, reteplase, urokinase, TNK-tPA		Avoid combination
PAR antagonist (atopaxar, vorapaxar)		Avoid combination
GPIIb/IIIa inhibitors (abciximab, eptifibatide, tirofiban)		Avoid combination
ADP receptor antagonists (cangrelor, clopidogrel, elinogrel, prasugrel, ticagrelor)		Monitor therapy
Thromboxane inhibitors:aspirin		Monitor therapy: aspirin < 100 mg is safe
Non-Steroidal Anti-inflammatory Agents: naproxen		Monitor therapy: naproxen < 500 mg per day is safe
Cyclooxygenase inhibitor (COX-2)		Monitor therapy
Cilostazol, dipyridamole, ticlopidine		Monitor therapy

Rifampin	Decrease efficacy via P-gp induction	Avoid combination
Carbamazepine, phenobarbital, primidone,		Monitor therapy
St. John's Wort (Hypericum Perforatum)		Monitor therapy
Tipranavir		Monitor therapy

Verapamil, trandolapril, quinidine	Enhanced bleeding via P-gp inhibition	Decrease the dose of edoxaban to 30 mg daily for VTE treatment. No dose adjustment required for atrial fibrillation (AF) (i) Verapamil: ↑ AUC of edoxaban by 52.7% (ii) Quinidine: ↑ AUC of edoxaban by 76.7%
Diltiazem		Dose reduction may be necessary for treatment of Venous Thromboembolism (VTE); and monitor therapy
Azithromycin, clarithromycin, erythromycin, amoxicillin, ketoconazole, itraconazole		Decrease the dose of edoxaban to 30 mg daily for VTE treatment. No dose adjustment required for AF
Posaconazole		Dose reduction may be necessary for VTE
Lansoprazole, omeprazole		Decrease the dose of edoxaban to 30 mg daily for VTE treatment. No dose adjustment required for AF
Amiodarone (oral administration at 600 to 1600 mg per day: causes inhibition of intestinal P-gp efflux pump inhibition), dronaderone		Dose reduction may be necessary for treatment of VTE; and monitor therapy (i) Amiodarone: ↑ AUC of edoxaban by 39.8% (ii) Dronaderone ↑ AUC of edoxaban by 84.5%
Propafenone		
Atorvastatin, lovastatin, simvastatin, niacin, ezetimibe, amlodipine, carvedilol, nicardipine, nifedipine, ralonazine		
Bosutinib, cabozantinib, crizitinib, lapatinib, nilotinib, regorafenib, pazopanib, sorafenib, tamoxifen, vemurafenib,		
Canagliflozin, metformin		Dose reduction may be necessary for treatment of VTE; and monitor therapy
Cobicistat, elvitegravir, emtricitabine, tenofovir, ritonavir, lopinavir, saquinavir, aimprevir, nelfinavir, lepidasvir, sofosbuvir, etravirine, draunavir, telaprivir, cyclosporine, eliglustat, enzalutamide, iloperidone, paliperidone, ivacaftor, mefloquine, mifepristone, pirfenidone, ulipristal, testosterone		
Grapefruit		
Tolvaptan		

**(b):** 

Interacting dietary supplements	Effect	Clinical Pearls

Thrombolytic activity: Nattokinase	Enhanced bleeding	
Decrease/inhibit platelet aggregation: caffeine, fish oil, fenugreek, flax seed, garlic, ginko, gingeng (panax, Siberian), willow bark, vitamin E (greater than 800 units per day), turmeric, resveratrol		
Anticoagulants: gamma Linolenic acid, glucosamine, melatonin,		Avoid combination
Warfarin derivatives: Alfalfa, celery seed, chamomile, dandelion, dong quai, horseradish, licorice root, horse chestnut, parsley, red clover, sweet clover, wild carrot, wild lettuce, nettle, passion flower, horseradish, cassia		

**Table 3:** Interruption/holding of edoxaban for procedures [17, 30, 31].

	*T*_1/2_	Low risk or minor surgery (procedures with 2-day risk for major bleeding 0–2%) ↓ Aiming for mild to moderate residual anticoagulant effect at surgery (12%–25%) or 2-3 half-lives	High risk or major surgery (procedures with 2-day risk for major bleeding 2%–4%) ↓ Aiming for no or minimal residual anticoagulant effect (3%–6%) at surgery or 4-5 half-lives

Edoxaban	10–14 hr	24 hr	48–72 hr

Types of surgical procedures		(i) Cholecystectomy (ii) Abdominal hernia repair (iii) Abdominal hysterectomy (iv) Coronary angiography/percutaneous coronary intervention (v) Electrophysiologic testing (vi) Pacemaker/cardiac defibrillator insertion (vii) Gastrointestinal endoscopy ± biopsy, enteroscopy, biliary/pancreatic stent without sphincterotomy, and endosonography without aspiration (viii) Minor plastic surgery (carpal tunnel repair) (ix) Minor orthopedic surgery/arthroscopy (x) Minor gynecologic surgery (dilation and curettage) (xi) Minor dental procedures (extractions) (xii) Minor skin procedures (cancer excision) (xiii) Minor eye procedures (cataract)	(i) Major cardiac surgery (surgical heart valve replacement/coronary artery bypass grafting) (ii) Major neurosurgical procedures (iii) Major cancer surgery (head and neck/abdominal/thoracic) (iv) Major orthopedic surgery (joint replacement/laminectomy) (v) Major urologic surgery (prostate/bladder resection) (vi) Major vascular surgery (vii) Kidney biopsy (viii) Polypectomy, variceal treatment, biliary sphincterectomy, and pneumatic dilation (ix) Endoscopically guided fine-needle aspiration

Creatinine clearance, CrCl.

**(a):** 

From	To	Recommendation
Warfarin	Edoxaban	Discontinue warfarin and start edoxaban when INR ≤2.5
Rivaroxaban, apixaban Dabigatran		Discontinue current oral anticoagulant and start edoxaban at the time of the next scheduled dose of the other oral anticoagulant
Low-molecular-weight heparin (LMWH) Fondaparinux		Discontinue LMWH or fondaparinux and start edoxaban at the time of the next scheduled administrastion of LMWH or fondaparinux
Heparin Intravenous Infusion (IVI)		Discontinue the infusion and start edoxaban 4 hours later

**(b):** 

From	To	Recommendations
Edoxaban	Warfarin	(1) Oral option: (i) For patients taking 60 mg of edoxaban, reduce the dose to 30 mg and begin warfarin concomitantly. (ii) For patients receiving 30 mg of edoxaban, reduce the dose to 15 mg and begin warfarin concomitantly. (iii) INR must be measured at least weekly and just prior to the daily dose of edoxaban to minimize the influence of edoxaban on INR measurements. (iv) Once a stable INR greater or equal to 2.0 is achieved, edoxaban should be discontinued and the warfarin continued. (2) Parenteral option: (i) Discontinue edoxaban and administer a parenteral anticoagulant and warfarin at the time of the next scheduled edoxaban dose. (ii) Once a stable INR greater or equal to 2.0 is achieved, the parenteral anticoagulant should be discontinued and the warfarin continued.
	Apixaban Rivaroxaban Dabigatran	Discontinue edoxaban and start the other anticoagulant at the time of the next dose of edoxaban.
	LMWH Fondaparinux Heparin IV	Discontinue edoxaban and start the parenteral anticoagulant at the time of the next dose of edoxaban.

**Table 5:** Summary of important edoxaban trials. [32–38].

Thromboprophylaxis after total knee replacement surgery					
Trial	Interventions	Duration (days)	*N*	Total VTE (%)	Major and CRNM bleeding (%)

STARS E-3 (Phase III)	Edoxaban 30 mg QD versus	11–14	716	7.4	6.2
	Enoxaparin 20 mg BID			13.9	3.7
				(*P* < 0.001 for NI)	(*P* = 0.129)

Thromboprophylaxis after total hip replacement surgery					
Trial	Interventions	Duration (days)	*N*	Total VTE (%)	Major and CRNM bleeding (%)

Phase IIb	Edoxaban	7–10	903	28.2 (*P* = 0.005)	3.7
	15 mg QD			21.2 (*P* < 0.001)	
	30 mg QD			15.2 (*P* < 0.001)	
	60 mg QD			10.6 (*P* < 0.001)	
	90 mg QD versus				
	Dalteparin 2,500 IU QD			43.5	6.2 (*P* = 0.129)
	followed by 5,000 IU QD				
Phase IIb	Edoxaban	11–14	264		
	15 mg QD			3.8	18
	30 mg QD versus			2.8	21.2
	Enoxaparin 20 mg BID			4.1	21.8
Phase III STARS J-V	Edoxaban	11–14	610		
	30 mg QD versus			2.4	2.6
	Enoxaparin 20 mg BID			6.9	3.7
				(*P* = 0.001 for NI)	

Thromboprophylaxis after hip fracture surgery					
Trial	Intervention	Duration (days)	*N*	Total VTE (%)	Major and CRNM bleeding (%)

STARS J-IV (Phase III)	Edoxaban	11–14	92		
	30 mg QD versus			3.7	3.4
	Enoxaparin 20 mg BID			6.5	6.9

Treatment and secondary prevention of VTE					
Trial	Interventions	Duration (months)	*N*	Total VTE (%)	Major and CRNM bleeding (%)

Hokusai-VTE (Phase III)	Enoxaparin or UFH/	3–12	8,292		
	Edoxaban 60 mg QD			3.2	8.5
	(or reduced to 30 mg QD) versus				
	Enoxaparin or UFH/			3.5	10.3
	warfarin (INR 2.0–3.0)			(*P* < 0.01 for NI)	(*P* = 0.004)

Prevention of stroke and systemic embolism in nonvalvular atrial fibrillation					
Trial	Interventions	Duration (years)	*N*	Annual rate of stroke and systemic embolism (%)	Annual rate for major bleeding (%)

ENGAGE-AF-TIMI 48	Edoxaban	2.8	21,105		
	60 mg QD or			1.18 (*P* < 0.001 for NI)	2.75 (*P* < 0.001)
	30 mg QD (or reduced to 15 mg QD)			1.61 (*P* = 0.005 for NI)	1.61 (*P* < 0.001)
	versus				
	Warfarin (INR 2.0–3.0)			1.50	3.43

*N*, number of patients; NI, noninferiority; QD, once daily; BID, twice daily; VTE, venous thromboembolism; CRNM, clinically relevant nonmajor; IU, international units; mg, milligrams; UFH, unfractionated heparin; INR, international normalized ratio.
